# Survival following radiotherapy in young women with localized early‐stage breast cancer according to molecular subtypes

**DOI:** 10.1002/cam4.2186

**Published:** 2019-04-23

**Authors:** Qi‐Qi Liu, He‐Fen Sun, Xue‐Li Yang, Meng‐Ting Chen, Yang Liu, Yang Zhao, Yuan‐Yuan Zhao, Wei Jin

**Affiliations:** ^1^ Department of Breast Surgery Key Laboratory of Breast Cancer in Shanghai, Fudan University Shanghai Cancer Center Shanghai China; ^2^ Department of Oncology Shanghai Medical College, Fudan University Shanghai China

**Keywords:** breast cancer, radiation therapy, molecular subtype, survival

## Abstract

**Background:**

To evaluate the significance and benefit of radiotherapy (RT) in young early‐stage breast cancer patients according to different molecular subtypes.

**Methods:**

We conducted a retrospective cohort study utilizing the Surveillance, Epidemiology, and End Results database with known hormone receptor (HoR) and human epidermal growth factor receptor 2 (HER2) status. Female patients aged 18‐45, received RT treatment, and diagnosed with stage T1‐3, N0‐3, M0 primary breast cancer between 2010 and 2013 were identified.

**Results:**

Of all the 23 148 included patients, 14 708 (63.54%), 3385 (14.62%), 1225 (5.29%), and 3830 (16.55%) were diagnosed with luminal‐A (HoR + HER2‐), luminal‐B (HoR + HER2+), HER2‐enriched (HoR‐HER2+), and triple‐negative (HoR‐HER2‐) breast cancer, respectively. RT was significantly correlated with improved overall survival (OS, HR: 0.295; 95% CI:0.138‐0.63, *P* = 0.002) and breast cancer‐specific survival (BCSS, HR: 0.328; 95% CI: 0.153‐0.702, *P* = 0.004) in HER2‐enriched patients. In addition, a significantly prolonged OS was also observed when RT was given to luminal‐A (HR: 0.696; 95% CI: 0.538‐0.902, *P* = 0.006) and luminal‐B (HR: 0.385; 95% CI:0.199‐0.744, *P* = 0.005) breast cancer patients compared to those without RT. Multivariable‐adjusted analyses showed that HER2 was a significant favorable factor for RT benefit in breast cancer patients.

**Conclusions:**

RT could offer significant survival benefit in luminal‐A, luminal‐B, and especially HER2‐enriched young early‐stage breast cancer female patients. The results enabled clinicians to predict the benefits of RT and improve evidence‐based treatment for breast cancer patients.

## 1 INTRODUCTION

Breast cancer is one of the most common cancers and the second leading cause of cancer death among women in the United States. Breast cancer is identified as having increased prevalence at younger ages and increased mortality rates for these years.^1^, ^2^ Approximately 11% of breast cancer women patients are diagnosed at age younger than 45 years.^3^ Compared with older counterparts, they are often accompanied by worse outcome, increased locoregional recurrence rate,^4^ and poorer treatment response owing to its more aggressive behaviors.^5^, ^6^ The Breast Cancer Education and Awareness Requires Learning Young (EARLY) act and prior research have identified woman under the age of 45 years to be particularly burdened by breast cancer.^7^, ^8^ In addition, researchers investigated that breast cancer women patients younger than 45 years are correlated with higher‐than‐expected frequencies of BRCA mutations, which are associated with an 50%‐85% elevated lifetime risk for breast cancer.^9^, ^10^ Radiotherapy (RT) could reduce recurrence risks, improve local control, and prolong overall survival (OS) which plays an indispensable role in the treatment for invasive breast cancer patients.^11^, ^12^

Breast cancer is classified into four major subgroups according to hormone receptors (HoR) and human epidermal growth factor receptor 2 (HER2) status, namely luminal‐A, luminal‐B, HER2‐enriched, and triple‐negative subtypes.^13^, ^14^ The heterogeneous of subgroups has substantial influence on survivals and recurrence risks of breast cancer. Studies have investigated correlations between subtypes and response to different treatment strategies including chemotherapy, targeted therapy, and endocrine therapy in breast cancer. However, despite the majority of young patients with localized early‐stage breast cancer receiving radiotherapy, the effect of HoR and HER2 status on benefit of RT in early‐stage breast cancer has not been integrated much.^15^ Thus, this is the first study comparing the RT benefit in young women with localized early stage breast cancer according to molecular subtypes, which could help predicting RT response and improve patient's survival by adjusting treatment strategies for individuals in the future.

## 2 MATERIALS AND METHODS

### 2.1 Ethics statement

The Surveillance, Epidemiology, and End Results (SEER) research data were obtained using the reference number 14684‐Nov2017. Informed consent is not required. Our study was in accordance with the ethical standards of Fudan University Shanghai Cancer Center (FDUSCC) and 1964 Helsinki Declaration. The methods were carried out according to the approved guidelines.

### 2.2 Study participants

We collected data of 23 148 patients using SEER*Stat (version 8.3.5), which reported cases from 18 population‐based registries (1973‐2013) on demographic characteristics including age, race, year of diagnosis, as well as laterality, grade, histology, TNM stage, ER, PR and HER2 status, tumor size, survivals and treatment strategies of patients in the United States. This analysis was limited to 18‐45 female patients, and diagnosed with stage T1‐3, N0‐3, M0 early‐stage breast cancer (American Joint Committee on Cancer [AJCC] stages I‐IIIC) between 2010 and 2013. The designation of “young women” with the 18‐45 age range was selected based on common clinical practices and previous researches.^16^, ^17^ All patients had complete information regarding the receipt of radiotherapy. Patients with ER and PR borderline were excluded for the accuracy of results. Patients were categorized according to their race (white, black, others, or unknown), age (18‐40 or 41‐45 years), laterality (left, right, bilateral, or unknown), tumor size (≤2 cm, 2‐5 cm, >5 cm, or unknown), breast‐adjusted AJCC sixth T (T1, T2, or T3), breast‐adjusted AJCC sixth N (N01, N1, N2, or N3), breast‐adjusted AJCC sixth stage (I, IIA, IIB, IIIA, or IIIC), grade (I, II, III, IV, or unknown), ER, PR, and HER2 status (positive or negative), and radiotherapy (yes or no). For molecular phenotyping, we defined HoR + as ER + and/or PR+, and HoR‐ as ER‐PR‐, while grouped tumors into four categories: luminal‐A (HoR+/HER2‐), luminal‐B (HoR+/HER2+), HER2‐enriched (HoR‐/HER2+), and triple‐negative (HoR‐/HER2‐).^18^, ^19^ Patients diagnosed before 2010 were excluded since the HER2 status and molecular subtype information were not available.

### 2.3 Statistical analysis

The clinicopathological characteristics among different subgroups were compared using the Pearson's *χ* ^2^ test. Breast cancer‐specific survival (BCSS) and OS were defined as the time from diagnosis to death due to breast cancer and any cause, respectively. Kaplan‐Meier analysis was performed to generate survival curves. Univariate and multivariate Cox hazard model was utilized to compare the prognostic role of RT in different subgroups, and calculate the HR and 95% CI. Subgroups were dichotomized according to the HoRs, HER2 status and molecular subtypes. Considering the characteristics unbalance between the subgroups, propensity score matching (PSM) was performed. All statistical analyses were performed using SPSS 25.0 software (SPSS, Chicago, IL). *P* < 0.05 was defined as statistically significant.

## 3 RESULTS

### 3.1 Patient characteristics by molecular subtypes

A total of 23 148 young female patients diagnosed with early‐stage breast cancer in 2010‐2013 were enrolled, including 16 966 white patients, 3041 black patients, and 2961 patients of other races. The demographic and clinicopathological characteristics are presented according to molecular subtypes in Table 1. The median age of patients was 41 years, 10 468 (45.2%) were younger than 40 years and 12 680 (54.8%) were 40‐45 years. Patients diagnosed with luminal‐A, luminal‐B, HER2‐enriched, and triple‐negative breast cancer were 14 708 (63.5%), 3385 (14.6%), 1225 (5.2%) and 3830 (16.5%), respectively. Radiotherapy was performed in 11 985 (51.8%) patients. The majority of patients received surgery (22 310, 96.4%), and nearly half (45.3%) were performed before RT. Eight thousand six hundred and sixty‐eight (37.4%), 7032 (30.4%), 3979 (17.2%), 2618 (11.3%), and 851 (3.7%) patients were diagnosed with stage I, IIA, IIB, IIIA, and IIIC, respectively. Nine thousand five hundred (41.0%) patients had positive lymph node metastasis. The median follow‐up was 22 months in the present study.

**Table 1:** Patient characteristics by molecular subtypes

Characteristics		Luminal‐A		Luminal‐B		HER2‐enriched		Triple negative		Total		*P* value
		(n = 14708)		(n = 3385)		(n = 1225)		(n = 3830)		(n = 23148)		
		N	%	N	%	N	%	N	%	N	%	
Race	White	10941	0.74	2473	0.73	871	0.71	2681	0.70	16966	0.73	**<0.001**
	Black	1677	0.11	415	0.12	179	0.15	770	0.20	3041	0.13	
	Others	1977	0.13	458	0.14	172	0.14	354	0.09	2961	0.13	
	Unknown	133	0.01	39	0.01	3	0.00	25	0.01	200	0.01	
Age	≤40	6018	0.41	1760	0.52	659	0.54	2031	0.53	10468	0.45	**<0.001**
	41‐45	8690	0.59	1625	0.48	566	0.46	1799	0.47	12680	0.55	
Year of diagnosis	2010	3506	0.24	819	0.24	298	0.24	931	0.24	5554	0.24	**0.035**
	2010	3787	0.26	786	0.23	309	0.25	1022	0.27	5904	0.26	
	2010	3686	0.25	851	0.25	319	0.26	917	0.24	5773	0.25	
	2010	3729	0.25	929	0.27	299	0.24	960	0.25	5917	0.26	
Laterality	Left	7302	0.50	1699	0.50	606	0.49	1970	0.51	11577	0.50	0.315
	Right	7405	0.50	1684	0.50	619	0.51	1858	0.49	11566	0.50	
	Bilateral	0	0.00	1	0.00	0	0.00	1	0.00	2	0.00	
	Unknown	1	0.00	1	0.00	0	0.00	1	0.00	3	0.00	
Differentiation	Grade I	2903	0.20	142	0.04	16	0.01	35	0.01	3096	0.13	**<0.001**
	Grade II	6811	0.46	1253	0.37	249	0.20	353	0.09	8666	0.37	
	Grade III	4486	0.31	1825	0.54	868	0.71	3270	0.85	10449	0.45	
	Grade IV	35	0.00	17	0.01	14	0.01	39	0.01	105	0.00	
	Unknown	473	0.03	148	0.04	78	0.06	133	0.03	832	0.04	
Histologic	Duct	11937	0.81	3072	0.91	1133	0.92	3506	0.92	19648	0.85	**<0.001**
	Lobular	1157	0.08	54	0.02	6	0.00	19	0.00	1236	0.05	
	Duct &lobular	882	0.06	121	0.04	13	0.01	45	0.01	1061	0.05	
	Others	732	0.05	138	0.04	4	0.00	260	0.07	1134	0.05	
TNM stage	I	6124	0.42	1123	0.33	383	0.31	1038	0.27	8668	0.37	**<0.001**
	IIA	4249	0.29	1017	0.30	341	0.28	1425	0.37	7032	0.30	
	IIB	2310	0.16	667	0.20	217	0.18	785	0.20	3979	0.17	
	IIIA	1564	0.11	445	0.13	195	0.16	414	0.11	2618	0.11	
	IIIC	461	0.03	133	0.04	89	0.07	168	0.04	851	0.04	
T	T1	8227	0.56	1584	0.47	557	0.45	1401	0.37	11769	0.51	**<0.001**
	T2	5283	0.36	1460	0.43	479	0.39	1972	0.51	9194	0.40	
	T3	1198	0.08	341	0.10	189	0.15	457	0.12	2185	0.09	
N	N0	8848	0.60	1843	0.54	627	0.51	2330	0.61	13648	0.59	**<0.001**
	N1	4355	0.30	1133	0.33	405	0.33	1098	0.29	6991	0.30	
	N2	1044	0.07	276	0.08	104	0.08	234	0.06	1658	0.07	
	N3	461	0.03	133	0.04	89	0.07	168	0.04	851	0.04	
Radiation	No	7507	0.51	1760	0.52	656	0.54	2062	0.54	11985	0.52	**0.01**
	Yes	7201	0.49	1625	0.48	569	0.46	1768	0.46	11163	0.48	
Radiation type	None	7507	0.51	1760	0.52	656	0.54	2062	0.54	11985	0.52	**0.001**
	Beam	7061	0.48	1608	0.48	557	0.45	1742	0.45	10968	0.47	
	Radioactive implant	62	0.00	4	0.00	3	0.00	5	0.00	74	0.00	
	Isotopes	0	0.00	0	0.00	0	0.00	2	0.00	2	0.00	
	Beam with implants/isotopes	11	0.00	3	0.00	0	0.00	3	0.00	17	0.00	
	Unknown	67	0.00	10	0.00	9	0.01	16	0.00	102	0.00	
Radiation sequence with surgery	None	7507	0.51	1760	0.52	656	0.54	2062	0.54	11985	0.52	**<0.001**
	Prior to surgery	29	0.00	11	0.00	9	0.01	15	0.00	64	0.00	
	Intraoperative	39	0.00	7	0.00	1	0.00	3	0.00	50	0.00	
	After surgery	7085	0.48	1593	0.47	546	0.45	1734	0.45	10958	0.47	
	Others	48	0.00	14	0.00	13	0.01	16	0.00	91	0.00	
Surgery	No	324	0.02	101	0.03	46	0.04	151	0.04	622	0.03	**<0.001**
	Yes	14279	0.97	3242	0.96	1162	0.95	3627	0.95	22310	0.96	
	Unknown	105	0.01	42	0.01	17	0.01	52	0.01	216	0.01	
Tumor size	≤2	8232	0.56	1588	0.47	560	0.46	1403	0.37	11783	0.51	**<0.001**
	2‐5	5318	0.36	1468	0.43	489	0.40	1980	0.52	9255	0.40	
	>5	1139	0.08	326	0.10	173	0.14	446	0.12	2084	0.09	
	Unknown	19	0.00	3	0.00	3	0.00	1	0.00	26	0.00	

P value was calculated among all groups by the Chi‐square test, and a bold type indicates significance.

Owing to the large sample size of our study, significant differences existed in clinical characteristics including race, age, differentiation, histologic, TNM stage, radiation type, surgery, and tumor size. Among the four molecular subgroups, luminal‐A patients presented with an older age (41‐45 years: 59.1% vs 48.0%, 46.2%, and 48.0% respectively; *P* < 0.001) and a lower differentiation degree (grade I: 19.7% vs 4.2%, 1.3% and 0.9%, respectively; *P* < 0.001). HER2‐enriched and triple‐negative patients were more likely to be grade III compared to luminal‐A and luminal‐B patients (70.8% and 85.4% vs 30.5% and 53.9%; *P* < 0.001). Luminal‐B and HER2‐enriched patients had more advanced tumors (tumor size >5 cm: 14.1% and 11.6% vs 7.7% and 9.6%, respectively; *P* < 0.001) than luminal‐A and triple‐negative patients. The incidence of lymph node metastasis (N3: 7.3% vs 3.1%, 3.9% and 4.4%, respectively; *P* < 0.001) and percentage of IIIA stage (15.9% vs 10.6%, 13.1% and 11.8%, respectively; *P* < 0.001) was higher in the HER2‐enriched patients than in the luminal‐A, luminal‐B and triple‐negative. No difference was found among subgroups at year of diagnosis and laterality.

### 3.2 Comparison of OS and BCSS among the study population

**Figure 1:**
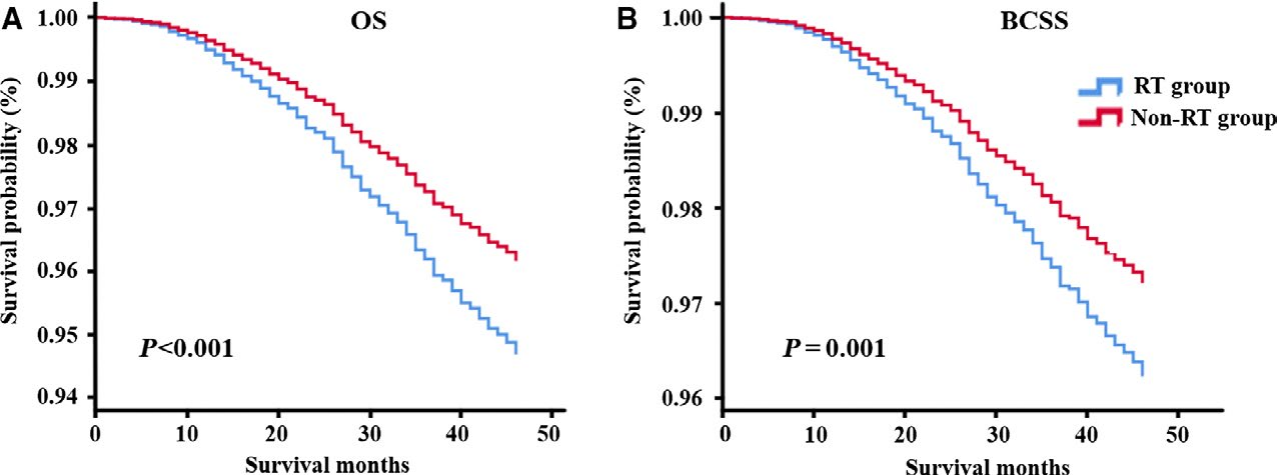
Kaplan‐Meier curve of overall survival (A) and breast cancer‐specific survival (B) in the whole early‐stage breast cancer patients

The Cox hazard models were conducted to evaluate effects of important characteristics on OS and BCSS in breast cancer patients. Without adjusting for confounding factors, univariate analysis indicated that black race, 41‐45 years of age, duct carcinoma, higher T and N stage, higher degree of differentiation and larger tumor size were associated with a worse overall survival, and a higher risk of death from breast cancer. However, age, T stage and tumor size were no longer distinctly correlated with prognosis in the adjusted multivariate model. Multivariate analysis results showed that RT was an independent prognostic factor for young early‐stage breast cancer patients. Compared to controlled groups, patients received RT had prolonged OS (HR: 0.717, 95% CI: 0.61‐0.844, *P* < 0.001) and BCSS (HR: 0.736, 95% CI: 0.617‐0.878, *P* = 0.001). The results were consistent with Kaplan‐Meier plots. In addition, black race, duct carcinoma, higher N stage, and differentiation degree were significantly associated with worse OS (all *P* < 0.05). All of the factors above were associated with higher breast cancer‐related mortality (*P* < 0.05) except duct carcinoma, which exhibited a borderline correlation with higher HR (*P* = 0.066). Results of survival analysis were summarized in Table 2, and Kaplan‐Meier curves of OS and BCSS by RT groups in the whole early‐stage breast cancer patients are shown in Figure 1.

**Table 2:** Comparison of overall survival (OS) and breast cancer‐specific survival (BCSS) among the study population

Characteristics		OS						BCSS					
		Univariate analysis			Multivariate analysis			Univariate analysis			Multivariate analysis		
		HR	95%CI	*P* value	HR	95%CI	*P* value	HR	95%CI	*P* value	HR	95%CI	*P* value
Race	White	ref.			ref.			ref.			ref.		
	Black	2.083	1.736‐2.50	**<0.001**	1.659	1.38‐1.993	**<0.001**	2.038	1.669‐2.488	**<0.001**	0.673	0.489‐0.927	**0.015**
	Others	0.685	0.51‐0.921	**0.012**	0.684	0.509‐0.92	**0.012**	0.68	0.494‐0.937	**0.018**	0.477	0.118‐1.921	0.298
	Unknown	0.747	0.24‐2.326	0.615	0.626	0.201‐1.955	0.421	0.556	0.138‐2.23	0.407	—		
Age	≤40	ref.			ref.			ref.			ref.		
	41‐45	0.753	0.644‐0.88	**<0.001**	0.989	0.844‐1.159	0.891	0.683	0.675‐0.81	**<0.001**	0.908	0.764‐1.080	0.275
Year of diagnosis	2010	ref.			ref.			ref.			ref.		
	2010	1.075	0.891‐1.297	0.448	1.092	0.905‐1.317	0.36	1.096	0.896‐1.341	0.371	1.114	0.91‐1.363	0.294
	2010	1.123	0.862‐1.462	0.391	1.093	0.838‐1.424	0.512	1.022	0.758‐1.377	0.888	0.974	0.722‐1.314	0.862
	2010	0.995	0.520‐1.905	0.988	1.01	0.527‐1.937	0.976	0.678	0.267‐1.724	0.415	0.676	0.265‐1.723	0.412
Laterality	Left	ref.			ref.			ref.			ref.		
	Right	0.96	0.821‐1.123	0.613	0.99	0.846‐1.158	0.898	0.91	0.768‐1.079	0.278	0.941	0.793‐1.116	0.483
	Bilateral	0.002	0‐3.16E + 63	0.938	0.004	0‐4.59E + 53	0.933	0.002	0‐5.11E + 66	0.94	0.001	0‐3.26E + 104	0.958
	Unknown	48.072	6.74‐343.06	**<0.001**	31.367	4.253‐231.4	**0.001**	55.977	7.835‐399.9	**<0.001**	47.927	6.397‐359.1	**<0.001**
Differen tiation	Grade I	ref.			ref.			ref.			ref.		
	Grade II	1.962	1.219‐3.159	**0.006**	1.553	0.962‐2.508	0.072	3.789	1.837‐7.815	**<0.001**	2.875	1.39‐5.945	**0.004**
	Grade III	6.843	4.373‐10.71	**<0.001**	4.142	2.624‐6.540	**<0.001**	14.897	7.4‐29.99	**<0.001**	8.529	4.205‐17.30	**<0.001**
	Grade IV	5.386	1.841‐15.76	**0.002**	3.534	1.204‐10.37	**0.022**	9.499	2.52‐35.806	**<0.001**	6.024	1.593‐22.79	**0.008**
	Unknown	6.742	3.881‐11.71	**<0.001**	4.737	2.711‐8.277	**<0.001**	12.831	5.809‐28.34	**<0.001**	8.533	3.839‐18.96	**<0.001**
Histologic	Duct	ref.			ref.			ref.			ref.		
	Lobular	0.476	0.294‐0.771	**0.003**	0.546	0.332‐0.899	**0.017**	0.475	0.279‐0.808	**0.006**	0.596	0.344‐1.034	0.066
	Duct &lobular	0.897	0.61‐1.32	0.582	1.154	0.781‐1.705	0.472	0.98	0.655‐1.465	0.921	1.298	0.864‐1.951	0.209
	Others	1.152	0.836‐1.588	0.388	1.239	0.896‐1.714	0.194	1.062	0.739‐1.528	0.744	1.215	0.842‐1.753	0.298
T	T1	ref.			ref.			ref.			ref.		
	T2	2.716	2.24‐3.293	**<0.001**	3.857	0.255‐58.29	0.33	3.174	2.545‐3.959	**<0.001**	2066.574	0‐6.10E + 22	0.739
	T3	5.985	4.796‐7.467	**<0.001**	2.234	0.342‐14.60	0.401	7.374	5.757‐9.446	**<0.001**	2.824	0.421‐18.94	0.285
N	N0	ref.			ref.			ref.			ref.		
	N1	1.791	1.483‐2.161	**<0.001**	1.407	1.158‐1.71	**0.001**	1.95	1.581‐2.405	**<0.001**	1.47	1.184‐1.825	**<0.001**
	N2	3.007	2.341‐3.861	**<0.001**	2.154	1.657‐2.802	**<0.001**	3.512	2.685‐4.594	**<0.001**	2.365	1.785‐3.133	**<0.001**
	N3	8.188	6.526‐10.274	**<0.001**	4.993	3.895‐6.401	**<0.001**	10.019	7.861‐12.77	**<0.001**	5.649	4.335‐7.361	**<0.001**
Radiation	No	ref.			ref.			ref.			ref.		
	Yes	0.982	0.84‐1.148	0.819	0.717	0.61‐0.844	**<0.001**	1.018	0.859‐1.206	0.84	0.736	0.617‐0.878	**0.001**
Tumor size	≤2	ref.			ref.			ref.			ref.		
	2 ~ 5	2.694	2.223‐3.265	**<0.001**	0.461	0.031‐6.922	0.576	3.13	2.511‐3.901	**<0.001**	0.001	0‐2.77E + 16	0.761
	>5	6.13	4.91‐7.654	**<0.001**	1.487	0.228‐9.712	0.678	7.574	5.913‐9.703	**<0.001**	1.316	0.197‐8.798	0.777
	unknown	3.833	0.536‐27.385	0.181	1.089	0.078‐15.29	0.95	4.891	0.683‐35.04	0.114	1.032	0.071‐14.95	0.982

The total CI and *P* value using Cox proportional hazards model and a bold type indicates significance. Abbreviations: HRs, hazard ratios; CI, confidence interval.

### 3.3 Survival analysis in matched groups according to HoR and HER2

Since clinicopathological characteristics imbalance existed in the study and RT were not randomly assigned, we performed PSM with a 1:1 nearest‐neighbor method according to HoR and HER2 status to further investigate their effects on the benefit of RT in patients. Kaplan‐Meier analysis was performed to compare survivals between patients with positive and negative HER2 status. As shown in Table 3, RT was an independent predictor for OS (HR: 0.343, 95% CI: 0.211‐0.558, *P* < 0.001) and BCSS (HR: 0.372, 95% CI: 0.218‐0.633, *P* < 0.001) in HER2 + breast patients. In addition, multivariate analysis demonstrated that RT was correlated with significantly prolonged OS and BCSS in the HER2 + patients. However, patients with HER2‐ received no benefit from radiotherapy in OS and BCSS in the adjusted multivariable models.

**Table 3:** Survival analysis in matched groups according to HER2

Characteristics		OS						BCSS					
		HER2‐			HER2+			HER2‐			HER2+		
		HR	95%CI	*P* value	HR	95%CI	*P* value	HR	95%CI	*P* value	HR	95%CI	*P* value
Race	White	ref.			ref.			ref.			ref.		
	Black	1.821	1.133‐2.925	**0.013**	1.712	0.98‐2.991	0.059	1.674	1.037‐2.702	**0.035**	1.299	0.659‐2.56	0.45
	Others	0.427	0.171‐1.065	0.068	1.397	0.739‐2.639	0.304	0.36	0.144‐0.898	0.028	1.584	0.807‐3.108	0.181
	Unknown	1.197	0.163‐8.788	0.859	2.343	0.317‐17.31	0.404	1.009	0.138‐7.38	0.993	0.005	0‐1.50E + 17	0.819
Age	≤40	ref.			ref.			ref.			ref.		
	41‐45	—			1.197	0.769‐1.863	0.425	1.301	0.657‐2.579	0.45	0.817	0.446‐1.498	0.514
Year of diagnosis	2010	ref.			ref.			ref.			ref.		
	2010	1.233	0.74‐2.02	0.432	0.769	0.441‐1.341	0.354	1.328	0.814‐2.167	0.256	0.817	0.446‐1.498	0.378
	2010	1.596	0.765‐3.331	0.213	0.759	0.362‐5.584	0.615	1.349	0.633‐2.879	0.438	0.598	0.215‐1.661	0.324
	2010	0.011	0‐1.50E + 10	0.754	1.421	0.362‐5.584	0.615	0.033	0‐6.755E + 9	0.797	0.627	0.073‐5.363	0.67
Laterality	Left	ref.			ref.			ref.			ref.		
	Right	0.811	0.535‐1.228	0.322	1.748	1.109‐2.755	**0.016**	0.799	0.53‐1.203	0.282	1.785	1.07‐2.978	0.026
	Bilateral	—			0.011	0‐1.47E + 85	0.965	—			0.01	0‐2.7E + 105	0.971
	Unknown	—			0.002	0	0.989	—			0.003	0	0.992
Differen tiation	Grade I	ref.			ref.			ref.			ref.		
	Grade II	0.935	0.379‐2.304	0.884	202.95	0‐4.128E + 10	0.586	0.939	0.323‐2.728	0.908	241.47	0‐9.32E + 11	0.626
	Grade III	2.004	0.838‐4.792	0.118	232.46	0‐4.714E + 10	0.577	1.995	0.712‐5.591	0.189	235.35	0‐9.06E + 11	0.628
	Grade IV	0.015	0‐3.11e + 12	0.802	365.22	0‐8.184E + 10	0.548	0.035	0‐1.705E + 7	0.742	480.36	0‐2.02E + 12	0.585
	Unknown	2.448	0.772‐7.764	0.128	385.06	0‐7.951E + 10	0.542	2.484	0.71‐8.689	0.154	209.67	0‐8.42E + 11	0.636
Histologic	Duct	ref.			ref.			ref.			ref.		
	Lobular	0.91	0.28‐2.96	0.875	1.127	0.154‐8.261	0.907	0.882	0.309‐2.52	0.815	1.317	0.179‐9.678	0.787
	Duct &lobular	2.156	1.068‐4.335	**0.032**	0.742	0.227‐2.43	0.623	1.899	0.897‐4.018	0.094	0.806	0.239‐2.714	0.728
	Others	0.814	0.296‐2.242	0.691	1.219	0.441‐3.372	0.702	0.86	0.314‐2.355	0.77	1.36	0.422‐4.387	0.607
T	T1	ref.			ref.			ref.			ref.		
	T2	1.496	0‐5.17E + 33	0.992	1.669	0‐6.908E + 40	0.991	0.711	0‐7.1533 + 40	0.994	1.785	0‐2.42E + 47	0.992
	T3	0.013	0‐1.56E + 27	0.899	0.005	0‐6.256E + 31	0.895	0.013	0‐4.57E + 37	0.926	0.006	0‐1.26E + 39	0.916
N	N0	ref.			ref.			ref.			ref.		
	N1	1.441	0.879‐2.363	0.148	1.316	0.728‐2.382	0.363	1.336	0.815‐2.188	0.251	1.015	0.48‐2.07	0.967
	N2	1.266	0.582‐2.755	0.552	3.371	1.672‐6.797	**0.001**	0.912	0.404‐2.059	0.825	3.289	1.512‐7.153	**0.003**
	N3	3.203	1.609‐6.375	**0.001**	10.38	5.379‐20.035	**<0.001**	3.129	1.604‐6.103	**0.001**	12.443	6.103‐25.37	**<0.001**
Radiation	No	ref.			ref.			ref.			ref.		
	Yes	1.129	0.725‐1.759	0.59	0.343	0.211‐0.558	**<0.001**	1.134	0.726‐1.77	0.581	0.372	0.218‐0.633	**<0.001**
Tumor size	≤2	ref.			ref.			ref.			ref.		
	2 ~ 5	1.221	0‐4.21E + 33	0.996	0.819	0‐3.383E + 40	0.997	3.467	0‐3.48E + 41	0.979	0.72	0‐9.72E + 46	0.995
	>5	301.53	0‐3.54E + 31	0.867	560.63	0‐7.091E + 36	0.874	407.006	0‐1.40E + 42	0.897	554.50	0‐1.92E + 44	0.896
	Unknown	0.566	0‐1.71E + 48	0.992	1.079	0‐1.529E + 84	0.999	1.331	0‐4.13E + 49	0.996	0.985	0‐8.1E + 107	1

The total CI and P value using Cox proportional hazards model and a bold type indicates significance. Abbreviations: HER2, human epidermal growth factor receptor 2; HRs, hazard ratios; CI, confidence interval.

Propensity score matching analysis was also conducted in HR status‐based subgroup. Results showed that in the multivariable models, patients underwent RT showed significant prolonged OS (HR: 0.693, 95% CI: 0.484‐0.993, *P* = 0.046) in HoR + subgroup, while no significant differences were observed in breast cancer‐specific mortality. In addition, RT‐treated patients showed better BCSS (HR: 0.773, 95% CI: 0.612‐0.978, *P* = 0.032) in patients in the HoR‐ cohort, while significant difference was not observed in OS between HoR + and HoR‐ subgroups. The results were shown in Table 4.

**Table 4:** Survival analysis in matched groups according to hormone receptors

Characteristics		OS						BCSS					
		HoR‐			HoR+			HoR‐			HoR+		
		HR	95%CI	*P* value	HR	95%CI	*P* value	HR	95%CI	*P* value	HR	95%CI	*P* value
Race	White	ref.			ref.			ref.			ref.		
	Black	1.461	1.134‐1.881	**0.003**	1.831	1.255‐2.672	**0.002**	1.449	1.113‐1.887	**0.006**	1.236	0.758‐2.017	0.395
	Others	1.07	0.743‐1.54	0.716	0.605	0.314‐1.166	0.133	0.964	0.652‐1.427	0.856	0.611	0.315‐1.186	0.146
	Unknown	0.001	0‐2.01E + 22	0.807	0.962	0.132‐7.024	0.969	0.001	0‐3.987E + 22	0.809	0.014	0‐144063.4	0.603
Age	≤40	ref.			ref.			ref.			ref.		
	41‐45	0.972	0.783‐1.208	0.801	1.116	0.806‐1.545	0.508	0.942	0.75‐1.183	0.608	0.915	0.61‐1.374	0.669
Year of diagnosis	2010	ref.			ref.			ref.			ref.		
	2010	0.959	0.745‐1.233	0.741	1.207	0.812‐1.796	0.353	0.955	0.735‐1.24	0.729	1.406	0.887‐2.23	0.147
	2010	0.907	0.63‐1.306	0.6	1.365	0.804‐2.317	0.25	0.884	0.601‐1.3	0.53	0.968	0.484‐1.936	0.926
	2010	0.727	0.283‐1.869	0.801	0.491	0.064‐3.751	0.493	0.674	0.235‐1.928	0.462	1.08	0.13‐8.99	0.943
Laterality	Left	ref.			ref.			ref.			ref.		
	Right	1.072	0.863‐1.332	0.528	0.813	0.588‐1.125	0.853	1.001	0.798‐1.256	0.993	0.81	0.551‐1.189	0.281
	Bilateral	0.001	0‐5.9E + 161	0.973	0	0	0.986	0.001	0‐2.1E + 166	0.973	0.019	0‐1.10E + 41	0.937
	Unknown	29.58	3.74‐233.9	**0.001**	0	0	0.998	32.01	4.00‐256.13	**0.001**	0.015	0	0.993
Differen tiation	Grade I	ref.			ref.			ref.			ref.		
	Grade II	3.078	0.413‐22.92	0.272	3070.0	0‐7.13E + 49	0.883	1186.90	0‐4.986E + 19	0.717	33.042	0‐1.212E + 9	0.694
	Grade III	2.939	0.402‐21.50	0.288	5094.8	0‐1.18E + 50	0.875	1099.73	0‐4.616E + 19	0.72	104.50	0‐3.786E + 9	0.601
	Grade IV	2.996	0.305‐29.04	0.21	0.532	0‐7.5E + 115	0.996	760.301	0‐3.272E + 19	0.734	67.21	0‐2.716E + 9	0.638
	Unknown	3.722	0.477‐29.04	0.21	7028.0	0‐1.63E + 50	0.871	1355.86	0‐5.711E + 19	0.712	139.41	0‐5.142E + 9	0.579
Histologic	Duct	ref.			ref.			ref.			ref.		
	Lobular	3.11	1.117‐8.654	**0.03**	0.586	0.229‐1.5	0.265	4.341	1.543‐12.22	**0.005**	0.392	0.091‐1.682	0.208
	Duct &lobular	1.912	0.971‐3.767	0.061	1.54	0.713‐3.324	0.271	2.091	1.065‐4.108	**0.032**	1.946	0.838‐4.522	0.121
	Others	0.966	0.624‐1.497	0.877	1.39	0.597‐3.234	0.445	1.042	0.658‐1.65	0.862	1.477	0.531‐4.111	0.455
T	T1	ref.			ref.			ref.			ref.		
	T2	5458.55	0‐4.862E + 31	0.793	0.662	0.014‐31.08	0.834	5740.50	0‐8.171E + 32	0.8	1.25	0‐8.06E + 36	0.996
	T3	2.007	0.267‐15.07	0.498	0.957	0.035‐26.16	0.979	1.699	0.222‐13	0.61	0.019	0‐7.27E + 32	0.922
N	N0	ref.			ref.			ref.			ref.		
	N1	1.652	1.257‐2.171	**<0.001**	1.31	0.876‐1.935	0.188	1.744	1.307‐2.327	**<0.001**	1.833	1.106‐3.038	**0.019**
	N2	3.215	2.272‐4.551	**<0.001**	2.325	1.375‐3.938	**0.002**	3.438	2.393‐4.940	**<0.001**	2.413	1.245‐4.676	**0.009**
	N3	5.574	3.98‐7.806	**<0.001**	4.752	0.176‐128.4	0.354	6.211	4.378‐8.811	**<0.001**	5.807	3.179‐10.61	**<0.001**
Radiation	No	ref.			ref.			ref.			ref.		
	Yes	0.835	0.666‐1.046	0.116	0.693	0.484‐0.993	**0.046**	0.773	0.612‐0.978	**0.032**	0.744	0.499‐1.108	0.145
Tumor size	≤2	ref.			ref.			ref.			ref.		
	2 ~ 5	0	0‐2.77E + 24	0.806	2.414	0.052‐112.2	0.653	0	0‐4.349E + 25	0.609	1.931	0‐1.24E + 37	0.988
	>5	1.365	0.182‐10.23	0.762	3.128	0.114‐85.74	0.5	1.7	0.223‐12.97	0.609	229.52	0‐8.96E + 36	0.894
	Unknown	0	0‐1.7E + 102	0.949	7.752	0.176‐127.4	0.354	0	0‐7.22E + 104	0.951	539.97	0‐2.16E + 37	0.877

The total CI and P value using Cox proportional hazards model and a bold type indicates significance. Abbreviations: HoR, hormone receptors; HRs, hazard ratios; CI, confidence interval.

### 3.4 Comparison of survival according to molecular subtypes

The multivariate model was utilized to identify OS and BCSS of breast cancer patients according to molecular subtypes. Results showed that RT was a significant predictor for favorable OS in luminal‐A (HR: 0.696, 95% CI: 0.538‐0.902, *P* = 0.006), luminal‐B (HR: 0.385, 95% CI: 0.199‐0.744, *P* = 0.005), and HER2‐enriched patients (HR: 0.295, 95% CI: 0.138‐0.63, *P* = 0.002). However, no significant benefit of RT on survivals was observed in the triple‐negative cohort (*P* = 0.534, Table 5).

**Table 5:** Comparison of overall survival (OS) according to breast cancer molecular subtypes

Characteristics		Luminal‐A			Luminal‐B			HER2‐enriched			Triple negative		
		HR	95%CI	*P* value	HR	95%CI	*P* value	HR	95%CI	*P* value	HR	95%CI	*P* value
Race	White	ref.			ref.			ref.			ref.		
	Black	1.795	1.343‐2.4	**<0.001**	1.677	0.786‐3.579	0.181	1.698	0.725‐3.98	0.223	1.263	0.97‐1.644	0.083
	Others	0.375	0.209‐0.674	**0.001**	0.608	0.183‐2.016	0.416	2.331	1.022‐5.318	**0.044**	0.923	0.607‐1.403	0.708
	Unknown	0.855	0.21‐3.481	0.502	3.06	0.398‐23.52	0.282	0.001	0‐2.5E + 209	0.977	0.002	0‐4.393E + 11	0.708
Age	≤40	ref.			ref.			ref.			ref.		
	41‐45	1.057	0.82‐1.363	0.667	0.75	0.33‐1.705	0.492	1.307	0.683‐2.499	0.419	0.864	0.685‐1.090	0.218
Year of diagnosis	2010	ref.			ref.			ref.			ref.		
	2010	1.305	0.959‐1.775	0.09	0.75	0.33‐1.705	0.492	0.702	0.321‐1.532	0.374	0.974	0.746‐1.272	0.847
	2010	1.464	0.96‐2.233	0.077	1.002	0.293‐3.427	0.998	0.627	0.194‐2.026	0.435	0.953	0.65‐1.397	0.805
	2010	1.26	0.427‐3.717	0.676	2.321	0.259‐15.01	0.376	0.945	0.104‐8.553	0.96	0.641	0.224‐1.834	0.407
Laterality	Left	ref.			ref.			ref.			ref.		
	Right	0.828	0.645‐1.064	0.14	2.02	1.068‐3.824	0.031	1.4	0.712‐2.751	0.329	1.016	0.808‐1.278	0.891
	Bilateral	—			0.008	0‐8.1E + 111	0.971	—			0.003	0‐8.454E + 87	0.957
	Unknown	0	0	0.999	0.003	0	0.993	—			23.141	2.873‐186.38	**0.003**
Differen tiation	Grade I	ref.			ref.			ref.			ref.		
	Grade II	1.086	0.645‐1.83	0.757	238.289	0‐2.37E + 12	0.641	666.716	0‐8.4e + 47	0.902	4.632	0.6‐35.767	0.142
	Grade III	3.257	1.982‐5.351	**<0.001**	257.683	0‐2.56E + 12	0.636	562.403	0‐7.07e + 47	0.905	3.534	0.467‐26.755	0.222
	Grade IV	0	0‐1.2E + 123	0.951	1143.473	0‐1.24E + 13	0.55	0.424	0‐5.91E + 63	0.991	3.813	0.378‐38.478	0.256
	Unknown	3.835	1.95‐7.542	**<0.001**	553.323	0‐5.63E + 12	0.591	487.493	0‐6.18E + 47	0.907	5.398	0.661‐44.077	0.116
Histologic	Duct	ref.			ref.			ref.			ref.		
	Lobular	0.525	0.286‐0.965	**0.038**	1.529	0.204‐11.48	0.68	0.004	0‐4.07E + 39	0.91	2.872	0.94‐8.772	0.064
	Duct &lobular	1.291	0.772‐2.159	0.331	1.003	0.234‐4.289	0.997	0.632	0.077‐5.177	0.669	2.384	1.155‐4.92	**0.019**
	Others	1.467	0.876‐2.456	0.145	1.468	0.343‐6.29	0.605	0.861	0.204‐3.639	0.839	0.941	0.592‐1.496	0.797
T	T1	ref.			ref.			ref.			ref.		
	T2	0.416	0.007‐26.13	0.678	1.187	0‐3.23E + 80	0.999	0.733	0‐1.38E + 64	0.997	2689.963	0‐9.236E + 29	0.8
	T3	0.927	0.024‐35.21	0.967	0.003	0‐8.42E + 62	0.94	0.002	0‐5.17E + 48	0.917	4.38	0.482‐39.764	0.189
N	N0	ref.			ref.			ref.			ref.		
	N1	1.31	0.968‐1.774	0.08	1.702	0.767‐3.779	0.191	0.921	0.369‐2.299	0.861	1.814	1.465‐2.411	**<0.001**
	N2	1.576	1.012‐2.454	**0.044**	3.458	1.217‐9.821	0.02	3.293	1.259‐8.614	**0.015**	3.467	2.384‐5.041	**<0.001**
	N3	4.038	2.682‐6.079	**<0.001**	12.918	5.107‐32.67	**<0.001**	7.688	2.94‐20.094	**<0.001**	6.335	4.419‐9.083	**<0.001**
Radiation	No	ref.			ref.			ref.			ref.		
	Yes	0.696	0.538‐0.902	**0.006**	0.385	0.199‐0.744	**0.005**	0.295	0.138‐0.63	**0.002**	0.927	0.73‐1.177	0.534
Tumor size	≤2	ref.			ref.			ref.			ref.		
	2 ~ 5	4.484	0.072‐279.2	0.477	1.16	0‐3.15E + 80	0.999	2.278	0‐4.29E + 64	0.991	0.001	0‐2.097E + 23	0.812
	>5	3.84	0.101‐146.5	0.469	759.254	0‐2.14E + 68	0.931	1571.243	0‐4.31E + 54	0.903	0.673	0.075‐6.049	0.724
	Unknown	3.888	0.102‐147.5	0.464	1.994	0‐8.2E + 168	0.997	1.732	0‐4.0E + 160	0.998	0.001	0	0.991

The total CI and P value using Cox proportional hazards model and a bold type indicates significance. Abbreviations: HRs, hazard ratios; CI, confidence interval.

**Figure 2:**
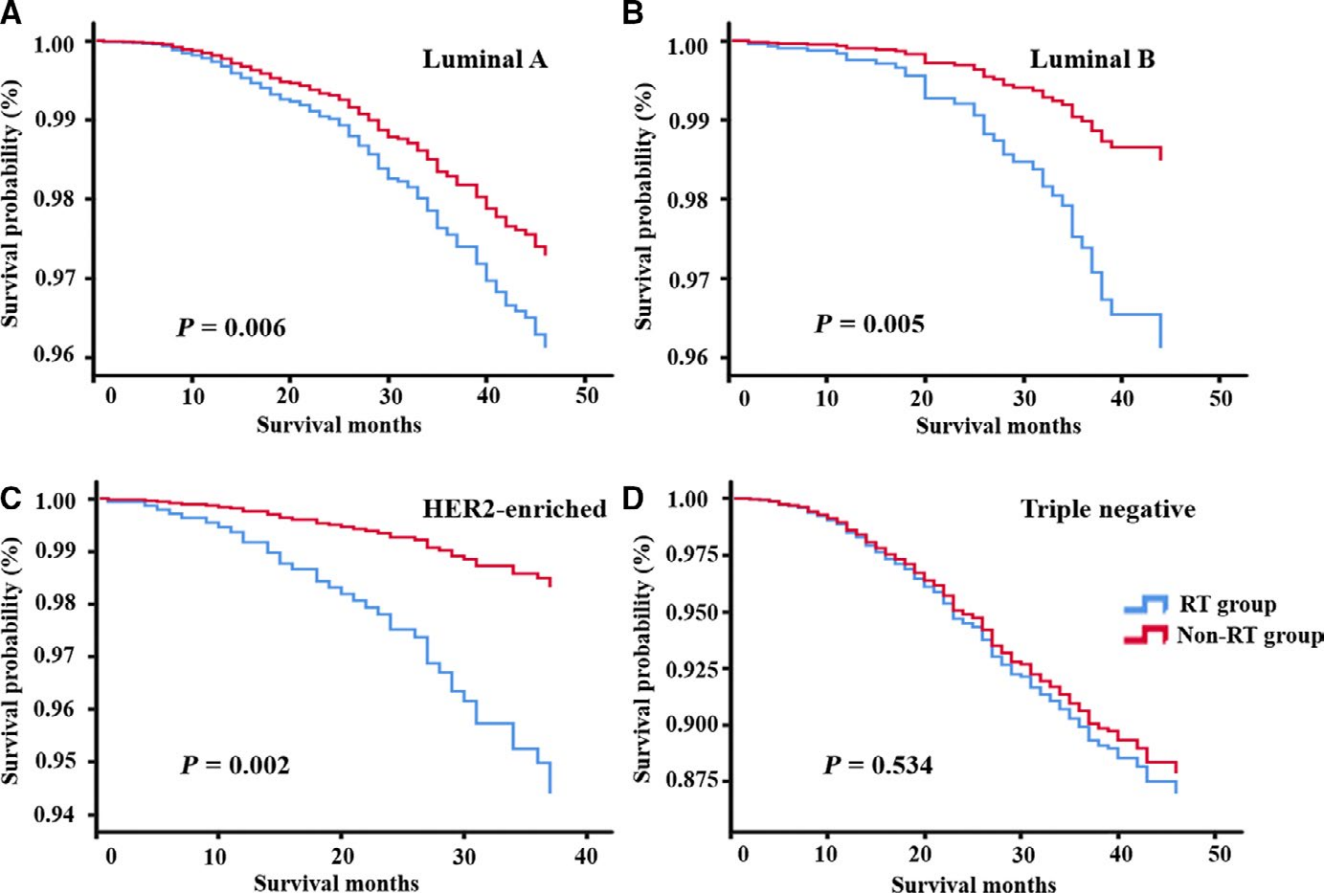
Kaplan‐Meier curve of overall survival in patients with luminal A (A), luminal B (B), HER2‐enriched (C) and triple negative (D) early‐stage breast cancer

**Figure 3:**
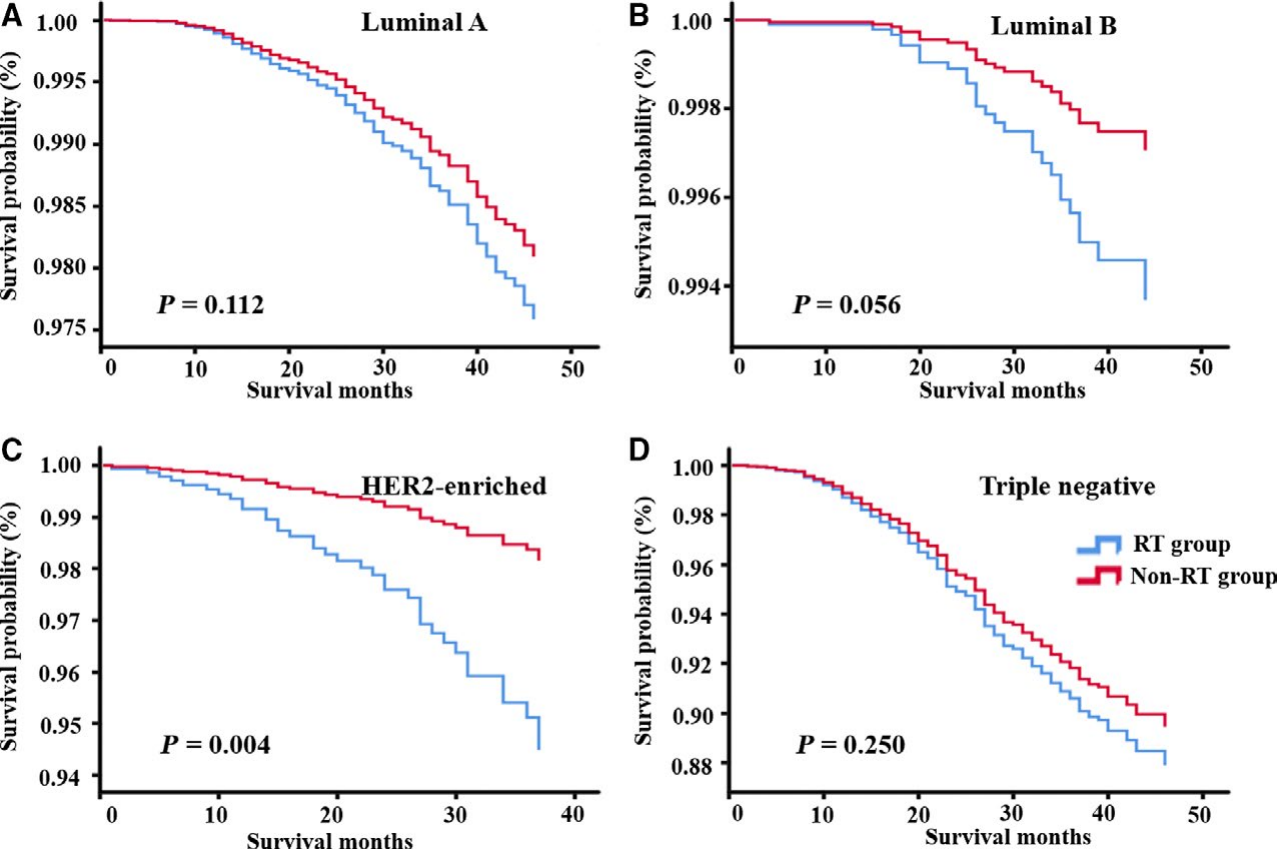
Kaplan‐Meier curve of breast cancer‐specific survival in patients with luminal A (A), luminal B (B), HER2‐enriched (C) and triple negative (D) early‐stage breast cancer

In the multivariate model for BCSS, RT was a significant independent prognostic predictor and correlated with prolonged survival in HER2‐enriched patients (HR: 0.328, 95% CI: 0.153‐0.702, *P* = 0.004). Notably, RT was associated with a slightly higher BCSS (HR: 0.464, 95% CI: 0.211‐1.020, *P* = 0.056) in the luminal‐B cohort but statistical significance was not reached. There was no significant difference in BCSS between RT‐treated patients and controlled patients in luminal‐A (*P* = 0.112) and triple‐negative patients (*P* = 0.250). Higher N stage was also an independent adverse prognostic factor for OS and BCSS in all breast cancer cohorts (all *P* < 0.05). Black race was associated with worse OS (HR: 1.795, 95% CI: 1.341‐2.4, *P* < 0.001) and BCSS (HR: 1.212, 95% CI: 01.212‐2.342, *P* = 0.002) in the luminal‐A cohort. Kaplan‐Meier curves of OS and BCSS for different breast cancer subgroups are shown in Figures 2 and 3, respectively.

### 3.5 Comparison of RT benefit according to the characteristics

**Figure 4:**
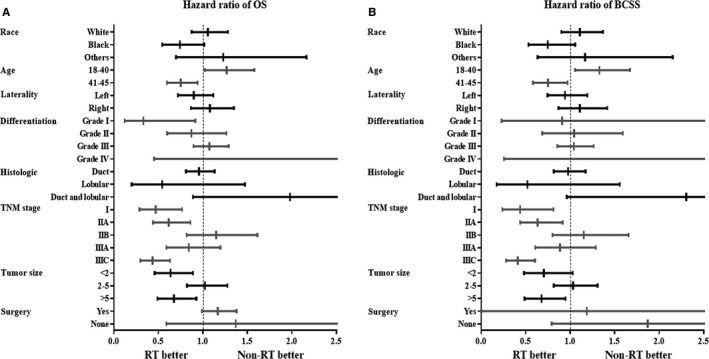
Hazard ratio and 95% confidence interval for overall survival (A) and breast cancer‐specific survival (B) according to the receipt of radiotherapy in the subgroup of patients for each characteristic

We conducted an exploratory subgroup analysis to identify potential benefit of RT in specific subgroups. The HRs and 95% confidence intervals comparing OS and BCSS according to the RT for several of characteristics were shown in Figure 4, and detailed statistics are listed in Supplementary Table S1. Results showed that response to radiotherapy for patients varies according to different characteristics and factors (Table 6).

**Table 6:** Comparison of breast cancer‐specific survival (BCSS) according to breast cancer molecular subtypes

Characteristics		Luminal‐A			Luminal‐B			HER2‐enriched			Triple negative		
		HR	95%CI	*P* value	HR	95%CI	*P* value	HR	95%CI	*P* value	HR	95%CI	*P* value
Race	White	ref.			ref.			ref.			ref.		
	Black	1.685	1.212‐2.342	**0.002**	0.728	0.214‐2.477	0.611	1.889	0.791‐4.510	0.152	1.234	0.936‐1.629	0.136
	Others	0.423	0.229‐0.783	**0.006**	0.56	0.13‐2.414	0.437	2.434	1.054‐5.624	**0.037**	0.780	0.49‐1.241	0.294
	Unknown	1.125	0.275‐4.605	0.87	0.033	0‐5.147E + 7	0.753	0.001	0‐3.37E + 220	0.978	0.002	0‐6.107E + 11	0.710
Age	≤40	ref.			ref.			ref.			ref.		
	41‐45	0.907	0.681‐1.21	0.507	0.996	0.457‐2.168	0.992	1.458	0.746‐2.85	0.27	0.816	0.638‐1.043	0.105
Year of diagnosis	2010	ref.			ref.			ref.			ref.		
	2010	1.395	0.989‐1.968	0.058	0.71	0.265‐1.901	0.496	0.80472697	0.36‐1.8	0.597	0.949	0.717‐1.255	0.711
	2010	1.236	0.742‐2.058	0.416	0.47	0.054‐4.078	0.494	0.682115	0.208‐2.235	0.528	0.910	0.605‐1.368	0.649
	2010	0.758	0.096‐5.963	0.792	0.018	0‐609481813	0.746	0.97835089	0.108‐8.894	0.984	0.545	0.163‐1.829	0.326
Laterality	Left	ref.			ref.			ref.			ref.		
	Right	0.792	0.597‐1.05	0.105	2.076	0.94‐4.586	0.071	1.492	0.737‐3.022	0.266	0.941	0.738‐1.199	0.621
	Bilateral	—			0.004	0‐1.024E + 213	0.982	—			0.003	0‐7.697E + 90	0.958
	Unknown	0	0	0.996	0.083	0	0.999	—			27.13	3.311‐222.234	**0.002**
Differen tiation	Grade I	ref.			ref.			ref.			ref.		
	Grade II	1.58	0.737‐3.39	0.24	101.652	0‐1126736963	0.577	790.066375	0‐9.042E + 53	0.911	635.9	0‐1.17E + 12	0.553
	Grade III	5.967	0.737‐3.39	0.24	77.579	0‐8.5667E + 8	0.588	639.936578	0‐7.309E + 53	0.914	473.1	0‐8.688E + 11	0.571
	Grade IV	0.009	0‐4.003E + 17	0.837	454.593	0‐5724106803	0.463	0.43039011	0‐4.647E + 70	0.992	380.4	0‐7.302E + 11	0.586
	Unknown	6.272	2.532‐15.537	**<0.001**	0.244	0‐3.156E + 19	0.952	572.751272	0‐6.597E + 53	0.916	676.4	0‐1.252E + 12	0.549
Histologic	Duct	ref.			ref.			ref.			ref.		
	Lobular	0.558	0.276‐1.129	0.105	1.811	0.238‐13.749	0.566	0.004	0‐2.095E + 40	0.839	4.094	1.304‐12.86	**0.016**
	Duct &lobular	1.45	0.834‐2.521	0.188	1.24	0.274‐5.617	0.78	0.735	0.088‐6.161	0.777	2.821	1.382‐5.75	**0.004**
	Others	1.19	0.624‐2.27	0.597	1.698	0.217‐13.285	0.614	0.953	0.225‐4.045	0.948	1.034	0.633‐1.688	0.894
T	T1	ref.			ref.			ref.			ref.		
	T2	299.262	0‐2.88E + 26	0.84	284.081	0‐2.002E + 100	0.961	0.68569615	0‐1.022E + 74	0.997	4456.1	0‐1.147E + 33	0.835
	T3	1.075	0.02‐56.525	0.972	0.97	0‐2.986E + 64	1	0.00167168	0‐1.158E + 60	0.931	3.084	0.325‐29.237	0.326
N	N0	ref.			ref.			ref.			ref.		
	N1	1.448	1.021‐2.053	**0.038**	1.142	0.382‐3.413	0.812	0.92105716	0.361‐2.352	0.863	1.941	1.435‐2.625	**<0.001**
	N2	1.79	1.101‐2.911	**0.019**	3.561	1.023‐12.391	**0.046**	2.75321208	0.994‐7.623	0.051	3.844	2.603‐5.678	**<0.001**
	N3	4.575	2.921‐7.168	**<0.001**	19.503	6.69‐56.856	**<0.001**	7.41758298	2.785‐19.754	**<0.001**	7.491	5.141‐10.915	**<0.001**
Radiation	No	ref.			ref.			ref.			ref.		
	Yes	0.789	0.589‐1.056	0.112	0.464	0.211‐1.020	0.056	0.328	0.153‐0.702	**0.004**	0.864	0.674‐1.109	0.250
Tumor size	≤2	ref.			ref.			ref.			ref.		
	2 ~ 5	0.007	0‐7.039E + 21	0.861	0.005	0‐3.232E + 95	0.963	2.45432835	0‐3.649E + 74	0.992	0.001	0‐8.679E + 26	0.847
	>5	3.875	0.073‐204.76	0.503	3.058	0‐9.404E + 64	0.988	1919.59015	0‐1.33E + 66	0.918	1.001	0.107‐9.357	0.999
	Unknown	4.385	0.084‐229.85	0.464	0.165	0‐1.998E + 137	0.991	1.30846106	0‐9.98E + 176	0.999	0.002	0	0.993

The total CI and P value using Cox proportional hazards model and a bold type indicates significance. Abbreviations: HRs, hazard ratios; CI, confidence interval.

Black patients showed prolonged OS (HR: 0.742 vs 1.058 and 1.229, all *P* > 0.05) and BCSS (HR: 0.749 vs 1.11 and 1.168, all *P* > 0.05) compared to white patients and others with RT treatment. Patients aged 41‐45 showed better benefit from RT on OS (HR: 0.751 vs 1.268, *P* = 0.014 and 0.031, respectively) and BCSS (HR: 0.752 vs 1.329, *P* = 0.029 and 0.016, respectively) compared to those younger than 40. In addition, breast cancer patients with grade I in differentiation degree was associated with prolonged OS, though not significant in BCSS after RT receipt. Patients diagnosed with I, IIA and IIIC showed better OS (HR: 0.469, 0.616 and 0.434, *P* = 0.002, 0.005 and <0.001, respectively) and BCSS (HR: 0.439, 0.634 and 0.411, *P* = 0.009, 0.016 and <0.001, respectively) received RT, while IIB and IIIA breast cancer showed no survival benefit from radiation treatment. A significantly prolonged OS was observed when RT was given to patients with breast tumor size less than 2 cm (HR: 0.639, *P* = 0.008), as well as larger than 5 cm (HR: 0.674, *P* = 0.015). Patients with tumor size larger than 5 cm could also benefit from RT in breast cancer‐specific survival (HR 0.679, *P* = 0.023).

## 4 DISCUSSION

Although RT has been accepted as one of the most significant treatments for breast cancer patients, the impact of molecular subtypes on RT response in early‐stage breast cancer patients has not been exactly elucidated in previous literatures.^20^, ^21^, ^22^, ^23^ To the best of our knowledge, this is the first population‐based retrospective study aimed to address the prognostic role of RT and its impact on OS and BCSS in young early‐stage breast cancer female patients according to molecular subtypes.

We carried out the Kaplan‐Meier analysis and found that RT is a significant predictor in the entire young early‐stage breast cancer cohort. Furthermore, we attempted to investigate the potential subgroups that would benefit from RT. Significant prolonged OS and BCSS were observed in RT‐treated HER2 + breast cancer patients compared to those with HER2‐ after demographic and clinicopathologic characteristics adjustment. However, patients treated with radiotherapy presented a significantly better OS in HoR + cohort, while a prolonged BCSS in the HoR‐subgroup. Multivariate analysis showed that relative to triple‐negative subtype, RT‐treated patients with luminal‐A, luminal‐B, or HER2‐overexpressing had a significant prolonged OS. HER2‐overexpressing breast cancer patients subjected to RT was also correlated with better BCSS.

Studies had investigated different effects of adjuvant RT according to tumor subtype in mastectomy cases. Marianne et al reported 1 000 high‐risk breast cancer patients received postmastectomy radiotherapy (PMRT) and demonstrated that HoR, HER2 status, and the constructed subtypes may be predictive of locoregional recurrence and survival. Results showed a significant improved OS among patients after PMRT characterized by good prognostic markers such as HoR + and HER2‐ patients.^24^ Another study demonstrated that triple‐negative patients had the highest risk of locoregional recurrence and the least benefit from PMRT, while the greatest effect was seen among luminal‐A patients.^25^ These researches indicated that the largest RT benefit was shown in luminal‐type breast cancer.

Luminal‐A and luminal‐B patients subjected to RT showed improved OS in our study, which was in consistent with the previous discoveries. We also revealed that HER2‐enriched patients showed the mostly benefit from radiation therapy. While a preclinical analysis revealed that no significant OS improvement after PMRT was found in HER2‐enriched high‐risk breast cancer patients,^24^ HER2 + was shown as a predictor for favorable survival compared with the HER2‐ in RT‐treated patients in our study. Furthermore, our results showed that RT was not an independent significant factor for BCSS in luminal‐A and luminal‐B breast cancer patients, with statistical significance not reached (*P* = 0.112 and 0.056, respectively). This discrepancy might be due to the following factors: (a) different inclusion criteria. Patients recruited in the previous study were presented with more malignancy‐associated properties, while patients were restricted to young early‐stage breast cancer in our study; (b) different therapy strategies. All their patients received a total mastectomy and a partial axillary dissection while surgery was given randomly in our study; (c) different types of radiotherapy. Apart from PMRT they performed, the type of RT in our study also included radioactive implant, isotopes. The sequence of RT with surgery also varies; (d) heterogeneity of patient's resources; and (e) the follow‐up period in our study was not long enough.

RT showed no significant prolonged OS and BCSS among patients with triple‐negative breast cancer (TNBC) in this study. Similar to our results, several of previous articles discovered that there might exist radio‐resistance in TNBC patients, which has not been confirmed though. A meta‐analysis performed by Kyndi et al showed that the local recurrence rates in triple‐negative patients did not decrease as much as those with luminal type, which indicated the relatively low radio‐sensitivity of TNBC cells.^24^ According to a research from The Cancer Genome Atlas, the radioresistance might be due to depletion or overexpression of several genes, such as the epidermal growth factor receptor (EGFR). In TNBC cells, EGFR overexpression could enhance proliferation and DNA damage response, as well as reduce apoptosis via PI3K‐Akt signaling pathway, and led to increased radioresistance.^26^ Other similar regulations including MELK overexpression or CDC27 depletion also contributed to the radio‐resistance of TNBC cells in the same way. However, all of these do not mean that RT in TNBC is not important. Studies clarified that postoperative radiotherapy for TNBC patients could reduce the local recurrence rate, especially for the patients with ≥4 positive axillary lymph nodes.^15^, ^27^, ^28^ In addition, we could discover and target specific potential biomarkers to regulate the proliferation and radio‐sensitivity of TNBC cells.

The underlying mechanism of how molecular subtypes affect RT benefit in breast cancer patients remains uncertain, further studies are still needed to elucidate potential signaling pathways. The results could help predicting tumor tissue response to RT, thus improve evidence‐based patients care and prolong patient's survival by adjusting therapy strategies. There also exist limitations in our study. First of all, we only included patients after 2010 since the HER2 status was not available before, which largely limited our follow‐up period. Secondly, some important information including Ki‐67 level, chemotherapy and endocrine therapy strategies were not available, this may result in potential bias. Lastly, the retrospective nature is an unavoidable weakness, thus larger perspective researches are needed to confirm the results. This limitation highlights the need for large designed prospective clinical researches to guide radiotherapy strategy in different IHC‐based subtypes of breast cancer, as well as achieve a longer follow‐up time to verify and extend our findings. In addition, therapeutic evaluation with systemic treatments including chemotherapy, hormone therapy and monoclonal target therapy should be added in further studies to understand RT benefit and the underlying mechanisms better. Despite the limitations, our study has several of strengths. No previous study has focused on the survival benefit in young early‐stage breast cancer for RT, though many have shown improvement in local control and local‐regional recurrence with postoperative radiation.^27^, ^28^ This is the first study comparing the RT benefit in young women with localized early stage breast cancer according to molecular subtypes via PSM to minimize potential bias. In addition, another major strength of this study is the large size of the patient cohort, which allowed us to provide contemporary information to the significance and benefit of RT in young early‐stage breast cancer patients according to different molecular subtypes‐that reflect the circumstances in the real world.

Based on our results, RT tended to have survival benefit in luminal‐A, luminal‐B and especially HER2‐enriched young early‐stage breast cancer female patients. In addition, HER2 was significant favorable factor for RT benefit in breast cancer patient. The results could help predicting RT response and improve patient's survival by adjusting treatment strategies for individuals. Further studies are needed to identify underlying mechanisms of the difference RT sensitivity in breast cancer molecular subtypes.

## ETHICS APPROVAL

Our study was in accordance with the ethical standards of Fudan University Shanghai Cancer Center (FDUSCC) and 1964 Helsinki Declaration.

## CONFLICT OF INTEREST

The authors declare no conflict of interest.
