# Supplementary material for: Survival following radiotherapy in young women with localized early‐stage breast cancer according to molecular subtypes
Source: Cancer Med. 2019 Apr 23;8(6):2840–57. doi: 10.1002/cam4.2186 (PMC6558475; doi:10.1002/cam4.2186)
Supplement: Supplementary file 1 [file CAM4-8-2840-s001.docx]

**Supplementary Table 1:** Hazard ratio and 95% confidence interval for OS and BCSS according to the receipt of radiotherapy in the subgroup of patients for each characteristic.

| Characteristic | | OS | | | BCSS | | |
| --- | --- | --- | --- | --- | --- | --- | --- |
|  |  | HR | 95%CI | P value | HR | 95%CI | P value |
| Race | White | 1.058 | 0.874-1.282 | 0.563 | 1.11 | 0.902-1.367 | 0.325 |
|  | Black | 0.742 | 0.543-1.014 | 0.061 | 0.749 | 0.532-1.056 | 0.099 |
|  | Others | 1.229 | 0.698-2.164 | 0.475 | 1.168 | 0.634-2.152 | 0.619 |
| Age | ≤40 | 1.268 | 1.022-1.576 | **0.031** | 1.329 | 1.055-1.673 | **0.016** |
|  | 41-50 | 0.751 | 0.598-0.943 | **0.014** | 0.752 | 0.583-0.972 | **0.029** |
| Laterality | Left | 0.898 | 0.72-1.119 | 0.338 | 0.943 | 0.744-1.196 | 0.63 |
|  | Right | 1.081 | 0.866-1.35 | 0.492 | 1.11 | 0.869-1.418 | 0.404 |
| Differentiation | Grade I | 0.333 | 0.121-0.916 | 0.033 | 0.911 | 0.2288-3.642 | 0.895 |
|  | Grade II | 0.871 | 0.6-1.264 | 0.468 | 1.045 | 0.686-1.592 | 0.838 |
|  | Grade III | 1.075 | 0.895-1.29 | 0.438 | 1.04 | 0.856-1.263 | 0.694 |
|  | Grade IV | 4.367 | 0.454-41.978 | 0.202 | 2.832 | 0.257-31.24 | 0.395 |
| Histologic | Duct | 0.958 | 0.81-1.133 | 0.614 | 0.979 | 0.816-1.175 | 0.823 |
|  | Lobular | 0.544 | 0.201-1.472 | 0.231 | 0.521 | 0.175-1.557 | 0.243 |
|  | Duct& lobular | 1.98 | 0.889-4.41 | 0.094 | 2.306 | 0.963-5.523 | 0.061 |
| TNM stage | I | 0.469 | 0.288-0.765 | **0.002** | 0.439 | 0.238-0.813 | **0.009** |
|  | IIA | 0.616 | 0.44-0.861 | **0.005** | 0.634 | 0.438-0.917 | **0.016** |
|  | IIB | 1.15 | 0.819-1.613 | 0.42 | 1.153 | 0.802-1.658 | 0.443 |
|  | IIIA | 0.842 | 0.592-1.198 | 0.339 | 0.885 | 0.609-1.287 | 0.523 |
|  | IIIC | 0.434 | 0.298-0.632 | **<0.001** | 0.411 | 0.279-0.604 | **<0.001** |
| Tumor size | ≤2 | 0.639 | 0.46-0.889 | **0.008** | 0.705 | 0.482-1.031 | **0.071** |
|  | 2~5 | 1.024 | 0.822-1.276 | 0.833 | 1.033 | 0.815-1.308 | 0.79 |
|  | >5 | 0.674 | 0.49-0.928 | **0.015** | 0.679 | 0.487-0.948 | **0.023** |
| Surgery | None | 1.37 | 0.589-3.187 | 0.465 | 1.872 | 0.793-4.424 | 0.153 |
|  | Yes | 1.168 | 0.989-1.38 | 0.068 | 1.188 | 0-14165.713 | 0.633 |
